# Potential impacts of Russo-Ukraine conflict and its psychological consequences among Ukrainian adults: the post-COVID-19 era

**DOI:** 10.3389/fpubh.2023.1280423

**Published:** 2023-09-28

**Authors:** Ahsan Riaz Khan, Ali Altalbe

**Affiliations:** ^1^Department of Interventional and Vascular Surgery, Shanghai Tenth People’s Hospital, Tongji University School of Medicine, Shanghai, China; ^2^National United Engineering Laboratory for Biomedical Material Modification, Branden Industrial Park, Qihe Economic and Development Zone, Dezhou, China; ^3^Department of Computer Engineering, Prince Sattam Bin Abdulaziz University, Al-Kharj, Saudi Arabia; ^4^Faculty of Computing and Information Technology, King Abdulaziz University, Jeddah, Saudi Arabia

**Keywords:** Russo-Ukraine conflict, COVID-19, coping mechanism, DASS-21, PSQI, depression, anxiety, stress

## Abstract

**Background:**

Since February 2022, the nation of Ukraine has become entangled in an escalating conflict that erupted after coronavirus outbreak fostering a situation of indeterminacy and precariousness, which adversely affected several facets, especially psychological well-being. However, there is a lack of empirical evidence on the psychological well-being of Ukrainians during the Russo-Ukraine war, as well as their coping strategies in response to the war’s repercussions. Consequently, this investigation endeavors to explore the prevalence of symptoms associated with depression, anxiety, stress, and insomnia and to correlate these symptoms with Ukrainians’ effective coping mechanisms during the ongoing war.

**Methods:**

An online survey was administered in Ukraine from June to August 2022 due to the ongoing Russo-Ukraine conflict. The survey employed a quota sampling technique, targeting 2,664 individuals (≥18 years). Out of the total sample, 1,833 valid responses were obtained, yielding a response rate of 68. 81%. Depression, anxiety, and stress were measured using the depression, anxiety, and stress scale-21 (DASS-21), while the Pittsburgh sleep quality index (PSQI) was utilized to evaluate insomnia symptoms. In addition, Brief-COPE was adopted to evaluate the coping mechanisms of the selected study participants.

**Results:**

Of 1,833 Ukrainian adults, 60.5% had symptoms of stress; 62.4% of them reported symptoms of anxiety; and 58.2% reported symptoms of depression. Symptom criteria for insomnia were found in about 21.8% of the study sample. The factors of sex, living area, area occupied by Russian forces, and having older adults and children in the house were statistically significant with symptoms of depression, anxiety, stress, and insomnia. The productive coping strategies of self-distraction, using instrumental support, planning, and behavioral disengagement, were observed as statistically significant with four psychological constructs.

**Conclusion:**

The study outcomes highlight a substantial prevalence of symptoms related to depression, anxiety, stress, and insomnia attributed to the accumulated consequences of ongoing conflict and the COVID-19 outbreak. The aforementioned findings emphasize the imperative of providing healthcare services and facilitating effective coping strategies among Ukrainians amid the ongoing war.

## Introduction

The impacts of warfare extend to both combatants and civilians, leading to detrimental physical and psychological manifestations for both groups. War can result in various deleterious physical consequences such as mortality, harm, sexual assault, starvation, diseases, and disabilities. Additionally, individuals exposed to war may experience post-traumatic stress disorder (PTSD), which further exacerbates the overall impact on their well-being. Depression and anxiety are among the emotional manifestations experienced by individuals (1, 2). The deleterious and alarming impact caused by the belligerence of armed conflict disrupts the lives of individuals and fractures relationships and families, thereby inducing profound emotional turmoil within affected individuals and communities (3).

The historical trajectory showcases the severe ramifications on national welfare resulting from armed conflicts (4). One of the consequential outcomes encompasses enduring physical and psychological detriments experienced by both children and adults. Numerous empirical investigations focusing on the broader populace have delineated a discernible rise in the occurrence and prevalence of mental afflictions stemming from armed conflicts. The confluence of the Russo-Ukrainian war with the ongoing global pandemic appears to exert an amplified impact on the well-being of Ukrainian individuals, particularly in terms of mental health (4). In light of the understanding that the healthcare system within Ukraine possessed considerable attributes, yet bore certain vulnerabilities, it can be inferred. During the prevailing coronavirus pandemic, which has notably imposed a substantial strain on healthcare systems and practitioners, such assaults possess the potential to generate even more severe consequences for Ukraine. According to data collected by the Surveillance System for Attacks on Health Care (SSA), a total of 157 instances of attacks on healthcare facilities were documented as of April 2022. The targeted assaults on crucial health infrastructure impede numerous individuals from accessing essential health services, despite their urgent and pressing health requirements. The impact of this traumatic experience is not solely confined to individuals who directly underwent it, but it can also be transmissible from parents to their subsequent offspring through nuanced yet inheritable modifications to the genome (5).

The present confluence of the Russo-Ukraine conflict and the prevailing coronavirus outbreak has fostered an exacerbation of the obstacles confronting the younger segment of the Ukrainian population (6). The ongoing pandemic that commenced in 2020 has potentially precipitated a heightened state of depression in individuals who are witnessing the warfare, consequently amplifying the preexisting psychological distress caused by the persisting threat of coronavirus. Hence, it was anticipated that there would be a positive correlation between individuals experiencing depression while observing the war and their perceived danger derived from the outbreak of coronavirus disease (7). The acquisition of weaponry among young males exposes them to more perilous and detrimental environments. Despite possessing robust immune systems, the coordinated efforts of these individuals present a potential danger to them. Young females are residing in densely populated shelters. Adolescents face an increased susceptibility during the process of flight, as they encounter the dismantling of their shielding interpersonal connections and encounter a lack of healthcare resources. The public health of individuals is at risk for both transmissible and non-communicable illnesses, as a consequence of diminished accessibility to medical care and resources, alongside diminished rates of inoculation and screening (1, 8).

Previous pieces of evidence have shown that exposure to terrorism and conflicts can have disastrous effects on the psychological well-being of individuals, leading to various mental health issues including depression, acute stress reactions, personality changes, cognitive impairments, anxiety disorders, and PTSD. This holds for those directly involved in the conflict such as combatants and veterans, as well as innocent civilians like refugees and the general population (9–12). The armed confrontation between Ukraine and Russia has already resulted in an assortment of psychological concerns (13, 14). An empirical investigation that evaluated the psychological well-being of the Ukrainian people prior to and following the recession discovered the proportion of people with indicators of stress increased by 5% from the year 2012 to 2016 (15). In connection with this, a survey conducted on 2,203 young Ukrainians who had been relocated across Ukraine in 2016 discovered that 32% of people had PTSD, 22% experienced depression, and 17% dealt with anxiety (10, 11).

As individuals undergo traumatic events and endure the stress of a prolonged war, there is a heightened likelihood of mental health issues and a decline in psychosocial well-being (16–18). However, there has been limited evidence conducted on the occurrence of psychological symptoms in the Ukrainian populace during the Russo-Ukraine conflict. According to scholarly research conducted in 2022, a notable incidence of war anxiety, reaching an approximate rate of 77.7%, was documented among the population of Polish citizens and Ukrainian refugees residing in Poland (19). A survey study was carried out among the student body and staff members of four universities in Ukraine, wherein a substantial occurrence of emotional states of anger, depression, nervousness, loneliness, and exhaustion was observed with a prevalence rate of 76.9, 84.3, 84.4, 51.8%, and 86. 7%, respectively (20). Hence, there arises a pressing need to investigate the incidence of mental illnesses within the broader populace amidst the Russian incursion into Ukraine.

The act of coping can be described as the conscious endeavors made to avert or mitigate threat, harm, and loss, or alleviate the ensuing anguish (21). Qualitative investigations have explored an array of coping mechanisms that Ukrainian individuals have embraced in response to Russia’s incursion. In a recent study, researchers conducted interviews with Ukraine refugees to investigate the various coping mechanisms employed to navigate their challenging circumstances. The findings indicated that establishing ongoing communication with their separated loved ones, actively acquiring accompaniment, and engaging in prayer emerged as the primary coping strategies (1, 2, 22). Additionally, *Khraban* analyzed discourses extracted from posts and comments shared on social media platforms by individuals residing in northern Ukraine throughout the initial 15-day period of the conflict. The research revealed a fluctuating utilization of coping mechanisms, specifically emotion-focused and problem-focused strategies, employed by civilians. These coping mechanisms served the purpose of expressing and alleviating negative emotions while simultaneously striving to mitigate the impact of distressing situations. Furthermore, they aimed to foster positive emotions such as benevolence and solidarity among the affected individuals (23).

The present study primarily focuses on the employed productive coping strategies, given the minimal mention of maladaptive coping methods involving substances like drugs and alcohol. This study aims to examine the efficacy of diverse coping strategies employed by the general populace of Ukraine in alleviating psychological symptoms amidst the ongoing conflict. The examination of coping strategies reveals categorization into two primary types: problem-focused and emotion-focused coping (24–26). In the realm of psychology, problem-focused coping pertains to the active endeavors made to eliminate the underlying problem at hand. Prior research has underscored the significance of problem-focused coping mechanisms in the context of managing wars or terrorism. In postwar Afghanistan, it was observed that Afghan individuals sought out enhanced sustenance, improved housing conditions, and augmented income levels (11). Emotion-focused coping pertains to efforts aimed at mitigating distress through the modification of problem perception or evaluation. This study aims to examine the impact of coping strategies employed during the Ukraine war. To procure a comprehensive and diverse collection of Ukrainian adults, we implemented a quota sampling method to explore their manifestation of depression, anxiety, stress, and insomnia, alongside their employed coping mechanisms.

## Methods

### Study design and population

Bearing in mind the ongoing conflict between Russia and Ukraine, prospective participants were sent invitations electronically, spanning from June to August 2022. The online survey was administered to attain a nationwide reach and ensure the secure participation of individuals through the utilization of mobile devices. To gather a representative sample of Ukrainian adults, we employed the non-probabilistic technique of quota sampling. This technique is widely recognized as a prominent technique for conducting online surveys in regions lacking access to a probabilistic panel (27, 28). In the present study, the utilization of quota sampling based on age, sex, and marital status proved to be a successful and feasible approach for procuring a representative sample of the Ukrainian population during the specified period. The survey was conducted utilizing the Ukrainian language and encompassed a cover page that provided a comprehensive explanation of the study’s scientific aims, ensuring that all participants provided their informed consent before commencing the survey. Out of the cohort of 2,664 adult individuals who were duly invited to partake in the examination, we have successfully procured a cumulative amount of 1,833 legitimate and relevant replies, yielding a commendable response rate of 68.81%.

### Ethics

The present survey received ethical approval from the Institutional Review Board of the university. In adherence to ethical principles, informed consent was duly procured from every participant involved in the investigation. Furthermore, stringent measures were adopted to safeguard anonymity and maintain strict confidentiality of the data acquired during the research process. No incentives were provided for the participants.

### Instruments

In this study, various socio-demographic attributes of the participants were collected encompassing age, sex, marital status, ethnic background, educational attainment, population of the respondents’ residential area, occupational status, presence of Russian forces in their locality, occurrence of active conflicts in the area, household size, as well as cohabitation of children and senior individuals within the same household.

### Depression, anxiety, and stress scale-21 (DASS-21)

The present study employed a set of 21 self-report questionnaires, specially crafted to evaluate the extent of severity of the fundamental symptoms associated with Depression, Anxiety, and Stress. This assessment tool has been previously validated and is widely acknowledged within the scholarly community (29). The administration of the survey may be carried out by the respondents themselves or facilitated by a third party. Every query must be addressed. The study utilizes a four-point Likert scale to ascertain the ratings of respondents, with each item assigned a numerical score ranging from *0* to *3*. The total score for each participant on the Depression, Anxiety, and Stress scales is obtained by multiplying the sum of seven pertinent items by two. Each scale is subsequently divided into subscales consisting of two to five items each.

### The Pittsburgh sleep quality index (PSQI)

The assessment of sleep quality (symptoms of insomnia) was conducted utilizing the PSQI, which comprehensively assesses sleep quality through the examination of 19 indicators categorized into seven distinct dimensions (30). The evaluated dimensions consist of subjective sleep disturbance, sleep quality, use of sleeping medication, sleep latency, sleep duration, sleep efficiency, and daytime dysfunction. In this study, a Likert scale with 4 levels was employed to assign scores ranging from 0 to 3 for each dimension, resulting in a total score between 0 and 21. A higher score on this scale indicates poorer sleep quality among the participants.

### The coping orientation to the problem experienced inventory (brief-COPE)

An adapted version of Brief-COPE was used to assess the coping mechanism of Ukrainian individuals (31). The questionnaire has been utilized in several investigations about conflict and is adequate for measuring coping mechanisms (32–34). The index measures 10 coping mechanisms using two items on a 7-point Likert scale, with 1 being strongly disagree and 7 being strongly agree.

The translation of all instruments was carried out in accordance with pertinent guidelines, in the Ukrainian language. Two autonomous translators were engaged in the process, wherein the adaptation encompassed a direct translation from English to Ukrainian language. This was followed by a reversed translation, and subsequently, psychological interviews were conducted with an initial set of participants. The reversed translated iteration was juxtaposed with the genuine rendition and any complications encountered throughout the process were deliberated upon and effectively tackled prior to the culmination of the conclusive rendition of the instruments.

### Statistical analysis

In order to gain insight into the respondents’ attributes and the prevalence ratio of symptoms related to mental well-being, an initial implementation of basic descriptive analyzes was conducted. Pearson’s correlation analysis was also performed to detect the multicollinearity among the study variables. Before we put the variables into the regression analysis, we checked for the assumptions. Shapiro–Wilk Test has been used to test the normality of the data. Subsequently, a series of multivariate analyzes were performed to evaluate the relationships between coping mechanisms and symptoms of mental well-being. The aforementioned cutoff rates were employed to categorize the four psychometric constructs as binary variables. In a parallel manner, logistic regression was executed to explore the association between coping strategies and mental health outcomes. We also tested the reliability of the constructs using Cronbach’s alpha in the present investigation. All the analyzes were performed using SPSS (v26) with a significant threshold of <0.05 and < 0.01.

## Results

Table 1 displays an overview of the sociodemographic characteristics of the study participants. According to the findings, the respondents exhibited an average age of 32.6 years, wherein a majority proportion of 61. 05% represented the female gender. The vast majority of the population comprises individuals affiliated with the Ukrainian ethnic group, accounting for approximately 98.85% of the total demographic composition. A total of 13.58% of the participants were found to reside in the region previously occupied by Russian forces, while 16.42% were identified as residing within the active conflict zone, as indicated within the context of the entire study sample. The findings indicate that a significant majority of the respondents, specifically 72.45%, reported residing within urban localities. Conversely, a remainder of 27.55% indicated their place of residence as rural regions. A majority of participants, comprising 59.7%, reported being married or in a cohabitating relationship. Following this, a substantial proportion of 35.7% indicated being single, while a smaller minority of 4.6% reported being divorced, separated, or widowed. The findings reveal that a majority of participants, constituting 75.99%, possessed a university degree or higher educational qualifications, while a smaller proportion of 24.01% were either students in school or college. Regarding the individual’s employment statuses, the majority of the participants constituted students (40.82%), followed by employees (39.72%), self-employed (81.3%), unemployed (64.9%), and pensioners (4.86%) Based on the statistical data, it can be observed that a significant proportion of households, amounting to 55.48%, consists of family sizes ranging from 3 to 5 members. The majority of participants indicated the presence of older individuals, such as parents or grandparents, residing within the same residence (56.46%) Meanwhile, a significant proportion of the sample acknowledged having children under the age of 18 coexisting within their household (43.54%).

**Table 1:** The social-demographic characteristics of study participants (*n* = 1833).

Demographic variables	*n* (%)	Significance
*Age*	32.6 ± 11.9	<0.001
*Sex*		0.04
Male	714 (38.95)	
Female	1,119 (61.05)	
*Marital status*		0.04
Single	654 (35.7)	
Married/cohabiting	1,094 (59.7)	
Divorced/separated/widowed	85 (4.6)	
*Ethnicity*		0.04
Ukrainian	1814 (98.85)	
Russian	19 (1.15)	
*Educational level*		<0.001
School/college level	440 (24.01)	
University and above	1,393 (75.99)	
*Location*		0.172
Urban	1,328 (72.45)	
Rural	505 (27.55)	
*The area occupied by Russian forces*		<0.001
Yes	249 (13.58)	
No	1,469 (80.15)	
Partially	115 (6.27)	
*Active fighting in the area*		<0.001
Yes	301 (16.42)	
No	1,446 (78.89)	
Do not know	86 (4.69)	
*Employment status*		0.01
Employed	728 (39.72)	
Unemployed	119 (6.49)	
Self-employed	149 (8.13)	
Student	748 (40.82)	
Pensioner	89 (4.86)	
*Household size*		0.03
1–2	101 (5.51)	
3–5	1,017 (55.48)	
6 or more	715 (39.01)	
*Children at home*		0.02
Yes	1,216 (66.34)	
No	617 (33.66)	
*Older adults at home*		0.02
Yes	1,035 (56.46)	
No	798 (43.54)	

Significance level at *p* < 0.05 and *p* < 0.001.

Table 2 displays the descriptive statistics and the analysis of the interrelationship among the four psychometric constructs. The study’s outcomes indicate that anxiety and stress were the predominant symptoms observed among the adult population in Ukraine. The average values of anxiety and stress were found to be 7.68 and 11.45, respectively, accompanied by standard deviations of 3.96 and 4.48. Notably, depression exhibited a mean of 9.26 and a prevalence rate of 58.2%. In contrast, the indications of insomnia were observed to be widespread, with a prevalence rate of 21.8%, accompanied by an average value of 12.11 and a standard deviation of 0.88. The bivariate analysis of the chosen study variables demonstrated a fundamentally symmetrical and unimodal distribution, indicating the absence of any non-linear relationships among the variables. The observed associations did not surpass the threshold of 0.7, indicating the absence of any discernible indications of multicollinearity.

**Table 2:** Descriptive statistics and bivariate correlation among psychometric constructs of the participants.

	Construct	Participants with symptoms *n* (%)	$\bar{X}\pm SD$	1	2	3	4	α
DASS-21								
1	Depression	1,066 (58.2)	9.26 ± 3.67	–				0.85
2	Anxiety	1,143 (62.4)	7.68 ± 3.96	0.463	–			0.84
3	Stress	1,108 (60.5)	11.45 ± 4.48	0.328	0.471	–		0.84
PSQI								
4	Insomnia	399 (21.8)	12.11 ± 0.88	0.356	0.392	0.502	–	0.86

DASS-21, Depression, anxiety, and stress scale 21; PSQI, Pittsburgh sleep quality index; $\bar{X}$ , Mean; SD, standard deviation; α, Cronbach’s alpha.

Table 3 represents the outcomes obtained from multivariate logistic regression analyzes conducted to explore the potential correlations between chosen demographic factors and coping strategies on psychometric indicators. The findings suggest that there was a higher likelihood of male participants displaying indications of stress, whereas female participants demonstrated a greater propensity for experiencing symptoms of anxiety, depression, and insomnia. Participants residing in urban regions displayed elevated levels of insomnia in comparison to individuals residing in rural regions. Individuals living with children reported a higher prevalence of insomnia, whereas those residing with older adults individuals displayed an elevated manifestation of depressive symptoms. One possible reason for this phenomenon could be attributed to the heightened vulnerability experienced by children and the older adults, who are regarded as particularly susceptible demographics during times of conflict. As a consequence, individuals coexisting with these individuals demonstrated a higher prevalence of mental health symptoms, potentially attributed to their additional obligations in providing care not only for themselves but also for their aforementioned counterparts. Individuals residing in regions under Russian occupation reported heightened levels of anxiety.

**Table 3:** Multivariate logistics regression analysis of demographic characteristics and coping strategies with psychometric constructs.

	DASS-21						PSQI	
	Depression	*p*-value	Anxiety	*p*-value	Stress	*p*-value	Insomnia	*p*-value
	B (SE)		B (SE)		B (SE)		B (SE)	
Age	5.64 (0.17)	<0.001	−6.12 (0.10)	<0.001	−5.46 (0.09)	<0.001	−3.28 (0.06)	<0.01
Sex	−0.39 (0.02)	0.128	0.79 (0.01)	0.072	1.19 (0.01)	0.207	2.01 (0.02)	0.074
Marital status	−0.41 (0.11)	0.173	0.91 (0.09)	0.185	0.21 (0.08)	0.199	−1.12 (0.07)	0.183
Ethnicity	1.23 (0.17)	0.343	−2.43 (0.12)	0.502	−1.14 (0.09)	0.086.		
Educational level	1.29 (0.07)	0.526	−1.07 (0.09)	0.418	−1.11 (0.07)	0.080	1.09 (0.08)	0.106
Location	1.09 (0.08)	0.117	1.82 (0.07)	0.612	2.13 (0.06)	0.293	3.45 (0.06)	0.112
The area occupied by Russian forces	−2.51 (0.12)	0.248	2.31 (0.17)	0.092	1.75 (0.81)	0.195	1.12 (0.71)	0.305
Active fighting in the area	−0.29 (0.24)	0.311	1.24 (0.18)	0.521	−0.76 (0.18)	0.439	1.13 (0.10)	0.181
Employment status	0.82 (0.05)	0.632	1.92 (0.11)	0.821	2.02 (0.05)	0.128	2.39 (0.04)	0.220
Household size	−1.19 (0.06)	0.761	1.37 (0.07)	0.093	0.78 (0.04)	0.405	2.28 (0.05)	0.543
Children at home	−0.88 (0.06)	0.192	−0.96 (0.12)	0.165	1.13 (0.09)	0.532	2.13 (0.06)	0.153
Older adults at home	−0.53 (0.11)	0.082	−0.63 (0.09)	0.323	3.18 (0.08)	0.452	−0.29 (0.20)	0.732
*Brief COPE*								
Adaptive coping								
Active coping	1.23 (0.07)	0.520	4.22 (0.04)	<0.05	2.16 (0.08)	<0.05	−2.29 (0.10)	0.093
Planning	1.68 (0.06)	0.074	2.61 (0.05)	0.521	2.93 (0.05)	0.603	4.14 (0.06)	0.543
Positive reframing	0.29 (0.05)	0.422	−0.73 (0.04)	0.428	−0.56 (0.04)	0.158	0.37 (0.04)	0.507
Humor	−1.16 (0.04)	0.119	1.34 (0.03)	0.106	1.78 (0.03)	0.217	−0.34 (0.04)	0.072
Acceptance	2.23 (0.05)	0.669	−2.18 (0.04)	0.354	−2.01 (0.04)	0.348	0.28 (0.11)	0.145
Religion	−2.17 (0.03)	0.105	2.43 (0.03)	0.532	−0.11 (0.04)	0.532	2.01 (0.03)	0.098
Use of emotional support	2.49 (0.09)	<0.05	−2.19 (0.07)	<0.01	−2.26 (0.07)	<0.01	−1.17 (0.10)	<0.01
Use of instrumental support	−3.41 (0.03)	<0.05	3.56 (0.02)	<0.01	2.16 (0.04)	0.088	2.07 (0.21)	0.191
Maladaptive coping								
Self-distraction	3.13 (0.06)	0.03	4.12 (0.04)	<0.01	2.69 (0.06)	<0.05	−3.19 (0.06)	<0.001
Denial	−1.46 (0.08)	0.165	−2.34 (0.09)	0.097	2.54 (0.08)	0.017	−2.13 (0.03)	0.429
Venting	−2.65 (0.05)	0.669	−3.47 (0.06)	0.532	−2.56 (0.04)	0.348	−3.42 (0.05)	0.165
Behavioral disengagement	−5.69 (0.02)	<0.001	2.83 (0.02)	0.087	3.18 (0.03)	<0.01	3.84 (0.10)	<0.01
*Pseudo* $R^{2}$	0.11		0.12		0.12		0.10	

DASS-21, Depression, anxiety, and stress scale 21; PSQI, Pittsburgh sleep quality index; S.E, standard error; α, Cronbach’s alpha.

Furthermore, the outcomes indicate a negative correlation between the utilization of instrumental support and behavioral disengagement coping strategies and the presence of stress symptoms. The utilization of self-distraction as a coping mechanism exhibited a negative correlation with symptoms of anxiety, depression, and insomnia while demonstrating a positive connection with symptoms of stress. There was a substantial linkage between active coping and anxiety symptoms, while a positive relationship was noted between planning and symptoms of insomnia. The utilization of instrumental support exhibited a positive correlation with anxiety symptoms. The findings of this study also demonstrate a substantial correlation between behavioral disengagement and the manifestation of depressive symptoms and sleep disturbances.

## Discussion

The present study represents the first comprehensive investigation of the associations between depression, anxiety, stress, insomnia, and coping strategies among Ukrainian citizens within a more diverse sample size. The advent of armed conflict generally poses a significant risk to the mental well-being of non-combatant individuals as a whole. In comparison with the incidence rates documented in 2016 regarding anxiety and depression (35), a comparatively higher proportion of Ukrainian adults experienced a diverse array of psychological manifestations, most notably depression, and anxiety (36). The obtained findings align with earlier research, which indicated a rise in the occurrence and frequency of psychological manifestations, among United States military veterans who have engaged in combat (37). Similarly, these results mirror the documented impacts of the Russo-Ukrainian discord on the mental well-being of the general population (15).

In response to the detrimental effects of the conflict, we further endeavored to explore the coping mechanisms embraced by Ukrainians and their correlation with the manifestation of psychological disorders. The results of our investigation indicate that the act of pursuing instrumental support exhibits a negative correlation with stress and a positive relationship with anxiety. This phenomenon may be attributed to the heightened propensity of individuals experiencing anxiety to actively solicit instrumental support. Based on empirical findings, it is apparent that instrumental social support coping emerged as a prominent coping mechanism employed by individuals residing in Israel in response to traumatic stress and the symptoms of PTSD after acts of terrorism. According to empirical research on the coping mechanisms used by Ukrainian military troops in counterterrorism missions, post-traumatic symptoms and the coping technique of soliciting social support have shown a negative correlation (38, 39).

Furthermore, our investigation revealed a positive correlation between active coping strategies and symptoms of anxiety. Similarly, we observed a positive association between planning strategies and symptoms of insomnia. Previous studies have reported that individuals tend to employ active coping and strategic planning mechanisms as a means of managing stressful situations. In another investigation it was discovered that Ukrainian students employ proactive coping mechanisms, specifically through the utilization of problem-solving planning, to navigate stressful situations successfully (40). Positive reframing was not observed to be correlated with symptoms linked to psychological well-being among Ukrainians in the early stage of the Russo-Ukraine conflict. A study conducted by Dickstein et al. (41) revealed that individuals residing in Israel employed a positive reframing strategy, namely, adopting an optimistic outlook on life, as a means to cope with stress induced by the persistent threat of terrorism. Another study demonstrated that the relationship between watching the war and depression is fully mediated by cognitive interference, suggesting that it is not the act of watching the war itself, but rather its connection to cognitive interference, that is linked to depression. There exists a positive relationship between denial, the act of refusing to acknowledge or accept the reality of the coronavirus threat, and depression (7). The lack of adoption and efficacy of this measure among Ukrainians can potentially be attributed to the rapid and intense escalation of the war, which may have hindered their ability to perceive it in a positive light.

Behavioral disengagement coping, characterized by the act of relinquishing efforts to cope with stressful situations, exhibited an unfavorable correlation with stress symptoms. However, its association with depression and insomnia was observed to be positive. The utilization of self-distraction coping strategies was found to have a positive correlation with indications of stress, yet a negative correlation with anxiety, depression, and insomnia symptoms. These outcomes are aligned with the results reported in previous investigations. In an investigation, it was observed that following the implementation of a threshold level of interventions, the adoption of avoidance and distraction methods was significantly associated with reduced post-Persian Gulf War stress responses (34). According to another research, it was found that engaging in activities to distract their thoughts proved to be beneficial for individuals displaced from Chechnya in managing their symptoms related to stress (42). Turetska (38) observed a noteworthy correlation between symptoms of PTSD and the tendency to employ problem-avoidance strategies in a cohort of Ukrainian military personnel. In a scholarly investigation conducted by researchers, it was identified that among a cohort of 3,294 adults, a specific coping strategy exhibited potential as a longitudinal mediator between major depressive disorder stemming from various traumatic events and generalized anxiety disorder (43). The outcomes of the present investigation also observed that emotion-focused coping mechanisms, along the lines of humor, religion, and seeking emotional support, displayed no apparent correlation with psychological symptoms among the Ukrainian population. The adverse circumstances resulting from war often engender a challenging environment wherein individuals find it arduous to engage in humor or derive amusement from the prevailing circumstances. The non-association between emotional support and religious coping, as identified in past conflicts to alleviate symptoms of mental illness such as anxiety, depression, or PTSD, was observed in the context of Ukraine, implying a lack of psychological symptom mitigation through these factors (11, 42, 44, 45).

The findings of our study indicate that there exists a positive association between the implementation of problem-focused coping mechanisms by Ukrainians and their mental health symptoms. Conversely, the effects of emotion-focused coping strategies displayed a varied pattern, displaying positive correlations with certain symptoms while demonstrating negative relationships with others. According to Hobfoll et al. (46) the implementation of emotion-focused coping strategies may yield significant effectiveness in handling highly demanding circumstances in the initial stages. Emotion-focused coping was discovered to be correlated with a subsequent rise in both the occurrence and intensity of post-traumatic symptoms among warriors over an extended period following combat engagement. The plurality of effects observed in our investigation necessitates further long-term investigations to elucidate the underlying causal associations between psychological disorders and emotion-focused coping mechanisms. However, the interconnectedness of naturally acquired coping mechanisms and psychological problems underscores the necessity of employing professional behavioral therapies in Ukraine.

Regrettably, the current repertoire of interventions provided by mental healthcare practitioners and centers in Ukraine frequently lacks a foundation in empirical evidence introducing a critical concern. According to a survey carried out in Ukraine in 2017, data suggests that mental illness has a prevalence rate of 33% within the population. However, a mere 4.9% of individuals grappling with such conditions are reportedly undergoing treatment (47). There is a substantial prevalence of psychological symptoms observed in Ukraine and Eastern Europe (48). Also, several obstacles impede individuals’ access to healthcare services, encompassing geographical remoteness, social stigma, feelings of shame, limited knowledge and awareness, substantial financial burdens associated with therapy, apprehension towards mental healthcare, and skepticism of the medical services. The ongoing coronavirus outbreak has already posed an immense burden on the healthcare network and healthcare personnel, rendering them susceptible to heightened stress levels. The Russian invasion has worsened this predicament, aggravating the existing challenges faced by the public healthcare network. Enhancing the professional development of physicians and psychologists by offering additional education and training in the field of mental health could potentially yield beneficial outcomes. Moreover, the provision of supplementary outreach services to individuals in Ukraine could potentially prove advantageous. The continuation of conflicts is probable, inevitably resulting in significant emotional anguish. Further exploration into empirical investigations is required in order to facilitate healing, foster resilience, and integrate cultural competence, with the aim of delivering suitable and efficacious mental health services to prospective war survivors.

## Limitations

Several limitations need to be recognized when it comes to the interpretation of the findings of the current study. The current study is grounded in a cross-sectional design conducted amidst the ongoing Russo-Ukraine conflict. Consequently, it is imperative to acknowledge that we are limited in our ability to establish a causal connection between psychological manifestations and coping mechanisms. It is conceivable that a circular association would exist between coping strategies and symptoms, wherein coping strategies exert an influence on symptoms, which subsequently instigate alterations in coping. There is a need for further investigation to elucidate the specifics of this association and establish the probable course of causation in prospective longitudinal investigations focusing on examining the links between symptoms of psychological manifestations and adopted coping mechanisms over an extended period. The present study directed its attention toward examining efficacious coping strategies. Strategies such as substance abuse, namely through drugs and alcohol, possess the potential to be counterproductive measures when attempting to handle the threat imposed by warfare. Subsequent inquiry may explore maladaptive coping mechanisms, such as substance and alcohol misuse, to ascertain their effects and potential interventions.

## Conclusion

An upsurge in psychological manifestations has been attributed to the simultaneous effects of the coronavirus outbreak and the Russo-Ukraine conflict. Given the prevalence of societal prejudices and restrictions, psychological wellness is frequently neglected in Ukraine; this propensity is exacerbated in times of conflict and epidemics, emphasizing the necessity for boosting essential backing for preventative services for emerging psychological demands and actions aimed at enhancing accessibility to care for ongoing and developing psychological disorders.

Our research offers preliminary insights regarding psychological health indicators such as depression, anxiety, stress, and insomnia amidst the upheaval as a consequence of the Russo-Ukraine invasion. Ukrainian individuals subjected to the Russian invasion have displayed a range of effective problem-solving coping mechanisms, including active coping and planning, in addition to adopting emotion-focused approaches such as seeking instrumental support, practicing behavioral disengagement, and resorting to self-distraction as means to mitigate these challenges related to psychological wellbeing. The aforementioned statistics highlight the imperative nature of providing expert medical care and psychotherapist services to individuals amidst times of invasion.

## Data availability statement

The raw data supporting the conclusions of this article will be made available by the authors, without undue reservation.

## Ethics statement

The study was conducted according to the guidelines of the Declaration and approved by the Ethics Review Committee of Prince Sattam Bin Abdulaziz, institutional review board, Deanship of scientific research (Ref: PS-04/25/2022). The studies were conducted in accordance with the local legislation and institutional requirements. Written informed consent for participation in this study was provided by the participants’ legal guardians/next of kin.

## Author contributions

AK: Conceptualization, Formal analysis, Investigation, Methodology, Writing – review & editing. AA: Funding acquisition, Investigation, Methodology, Resources, Writing – original draft, Writing – review & editing.

## Funding

The author(s) declare financial support was received for the research, authorship, and/or publication of this article. The authors extend their appreciation to Prince Sattam bin Abdulaziz University for funding this research work through the project number (PSAU/2023/01/224253).

## Conflict of interest

The authors declare that the research was conducted in the absence of any commercial or financial relationships that could be construed as a potential conflict of interest.

## Publisher’s note

All claims expressed in this article are solely those of the authors and do not necessarily represent those of their affiliated organizations, or those of the publisher, the editors and the reviewers. Any product that may be evaluated in this article, or claim that may be made by its manufacturer, is not guaranteed or endorsed by the publisher.
